# Transplantation of patient‐specific bile duct bioengineered with chemically reprogrammed and microtopographically differentiated cells

**DOI:** 10.1002/btm2.10252

**Published:** 2021-09-03

**Authors:** Elina Maria Buisson, Suk‐Hee Park, Myounghoi Kim, Kyojin Kang, Sangtae Yoon, Ji Eun Lee, Young Won Kim, Nak Kyu Lee, Mi Ae Jeong, Bo‐Kyeong Kang, Seung Bum Lee, Valentina M. Factor, Daekwan Seo, Hyunsung Kim, Jaemin Jeong, Han Joon Kim, Dongho Choi

**Affiliations:** ^1^ Department of Surgery Hanyang University College of Medicine Seoul Republic of Korea; ^2^ HY Indang Center of Regenerative Medicine and Stem Cell Research Hanyang University Seoul Republic of Korea; ^3^ School of Mechanical Engineering Pusan National University Busan Republic of Korea; ^4^ Digital Manufacturing Process Group Korea Institute of Industrial Technology Siheungsi Gyeonggi‐do Republic of Korea; ^5^ Department of Anesthesiology and pain medicine Hanyang University College of Medicine Seoul Republic of Korea; ^6^ Department of Radiology Hanyang University, College of medicine Seoul Republic of Korea; ^7^ Laboratory of Radiation Exposure & Therapeutics National Radiation Emergency Medical Center, Korea Institute of Radiological & Medical Science Seoul Republic of Korea; ^8^ Laboratory of Molecular Pharmacology Center for Cancer Research, National Cancer Institute, National Institutes of Health Bethesda Maryland USA; ^9^ Psomagen Inc Rockville Maryland USA; ^10^ Department of Pathology Hanyang University College of Medicine Seoul Republic of Korea; ^11^ Present address: Current address: School of Mechanical Engineering Purdue University West Lafayette Indiana USA

**Keywords:** 3D printing, chemically derived hepatic progenitors, cholangiocytes, electrospun fibers, patient‐specific bile duct

## Abstract

Cholangiopathy is a diverse spectrum of chronic progressive bile duct disorders with limited treatment options and dismal outcomes. Scaffold‐ and stem cell‐based tissue engineering technologies hold great promise for reconstructive surgery and tissue repair. Here, we report a combined application of 3D scaffold fabrication and reprogramming of patient‐specific human hepatocytes to produce implantable artificial tissues that imitate the mechanical and biological properties of native bile ducts. The human chemically derived hepatic progenitor cells (hCdHs) were generated using two small molecules A83‐01 and CHIR99021 and seeded inside the tubular scaffold engineered as a synergistic combination of two layers. The inner electrospun fibrous layer was made of nanoscale–macroscale polycaprolactone fibers acting to promote the hCdHs attachment and differentiation, while the outer microporous foam layer served to increase mechanical stability. The two layers of fiber and foam were fused robustly together thus creating coordinated mechanical flexibility to exclude any possible breaking during surgery. The gene expression profiling and histochemical assessment confirmed that hCdHs acquired the biliary epithelial phenotype and filled the entire surface of the fibrous matrix after 2 weeks of growth in the cholangiocyte differentiation medium in vitro. The fabricated construct replaced the macroscopic part of the common bile duct (CBD) and re‐stored the bile flow in a rabbit model of acute CBD injury. Animals that received the acellular scaffolds did not survive after the replacement surgery. Thus, the artificial bile duct constructs populated with patient‐specific hepatic progenitor cells could provide a scalable and compatible platform for treating bile duct diseases.

## 1 INTRODUCTION

Cholangiopathy, a syndrome of biliary obstruction and liver damage, represents a diverse spectrum of biliary tract diseases of different etiology frequently affecting multiple organs and making it a complex target for therapy. The treatment prospects are limited without proven therapeutic effects with whole liver transplantation remaining the primary therapeutic option.^1^ However, the demand for transplantation is greatly exceeding the availability of healthy donor liver, besides the postoperative complications such as immune rejection and infections increase the risks of morbidity and mortality.^2^

Among the breakthrough applications in the management of the common bile duct (CBD) disorders is the generation of bioengineered bile ducts.^3^, ^4^, ^5^, ^6^, ^7^ More recent techniques allow for the production of tridimensional (3D) tubular scaffolds in combination with cells and biologically active molecules to form functional bile duct structures.^2^, ^8^ A variety of techniques, biomaterials, and cell types, including primary cholangiocytes^5^ and stem cells,^6^ have been adopted to create 3D scaffolds promoting cell adhesion and proliferation, and possessing mechanical strength sufficient for transplantation.^9^, ^10^, ^11^ However, the tridimensionality in most of the previous works still stayed at the level of simple cylindrical shape. The 3D scaffolds would need a further development toward 3D customization with arbitrary shapes for patient‐specific clinical needs.

Here, we introduce a novel combinatorial bioengineering approach to build a new type of artificial bile duct (ABD) for medical applications. For the 3D customizable shaping, the fabrication method is utilizing our recently published technique for scaffold fabrication using medical image‐based 3D printing of a sacrificial template and dip coating of biomaterials.^12^ To improve cell‐to‐matrix topographic cues and increase the mechanical stability, the tubular scaffold was formed as a bilayer construct of a microscale fibrous mat surrounded by a microporous foam layer that reinforced the structure while providing mechanical flexibility. For epithelization of the artificial tubular scaffold, we utilized human chemically derived hepatic progenitor cells (hCdHs) as a compelling cell source of patient‐specific stem cells amenable to self‐renewal and differentiation toward biliary epithelial lineage.^13^

## 2 RESULTS AND DISCUSSION

### 2.1 Fabrication of 3D customized bilayer tubular scaffold

**FIGURE 1:**
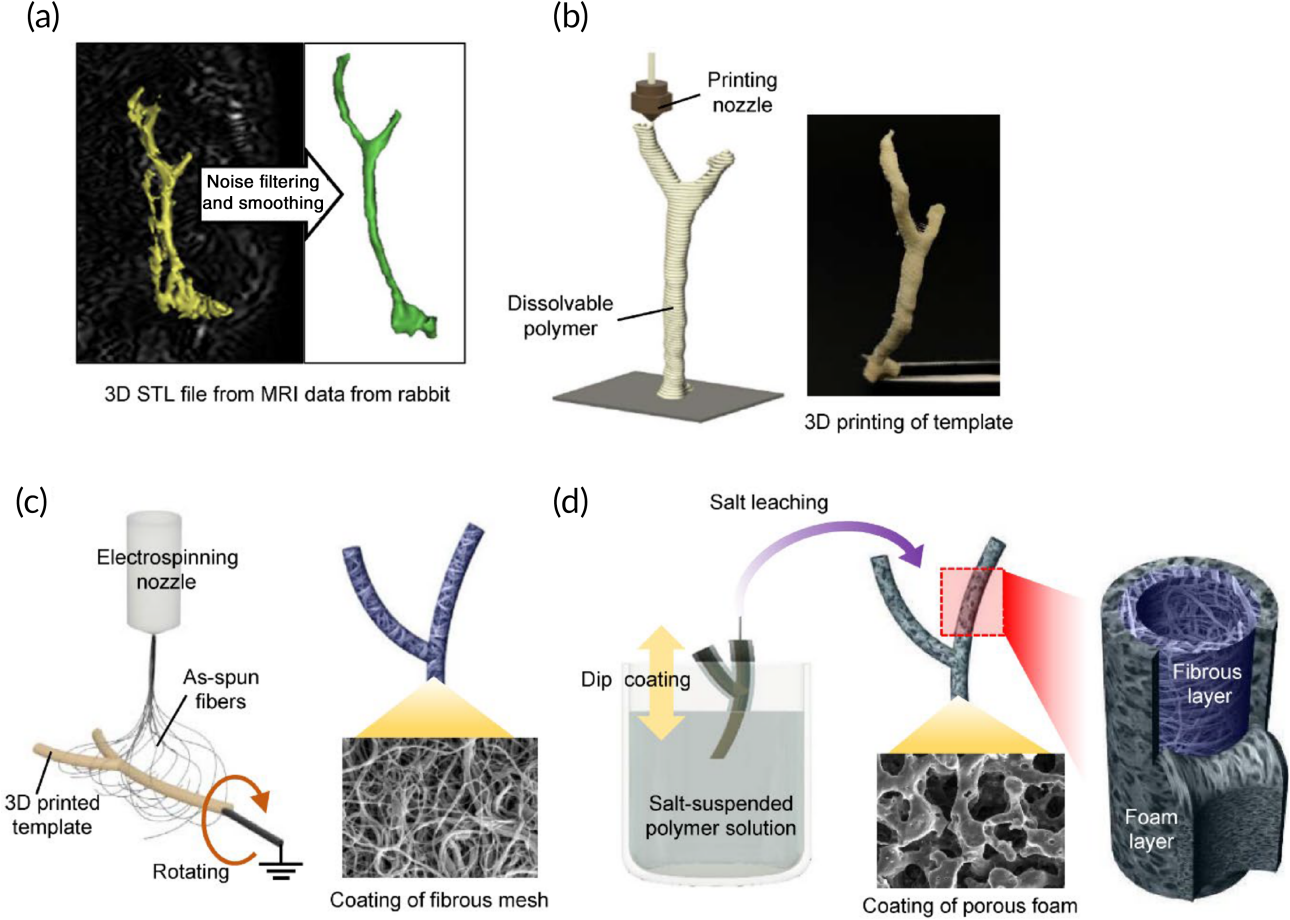
Overall procedures for bioengineering of 3D patient‐specific cell‐laden artificial bile duct construct. (a) 3D design of target bile duct on the basis of magnetic resonance imaging (MRI) data. (b) Fabrication of water‐soluble sacrificial template using material extrusion‐based 3D printing process according to 3D model data. (c) Inner layer formation of fiber mat by electrospinning onto 3D printed template. (d) Outer layer formation of porous foam by dip coating in salt‐suspended polymer solution and salt leaching

To create a personalized rabbit‐size living construct resembling the native CBD, we utilized our recently published approach based on 3D design, 3D printing, postprocessing, and animal experimentation.^12^ The fabrication of 3D customized tubular scaffold involved computer‐aided design (CAD) based on the magnetic resonance imaging data. From the 2D magnetic resonance cholangiopancreatography images, the target object in biliary tree was three‐dimensionally reconstructed and postprocessed as a 3D printable file format (STL) (Figure 1a). According to the 3D model data, a sacrificial template was then fabricated using a water‐soluble polymer polyvinylalcohol (PVA) in accordance with a 3D printing method of material extrusion (Figure 1b). To provide a low‐stiffness microenvironment supporting cell adhesion and proliferation, a hybrid bilayer film was formed on the surface of the PVA template replacing a simple monolayer used in our earlier publication.^12^ To create a fibrous topography of the inner surface of the first layer, electrospinning was performed on the rotating template thereby depositing the as‐spun fibrous mat on the circumference of the template (Figure 1c). For the second layer, the fiber‐deposited template was dip‐coated in salt‐suspended polyurethane solution followed by leaching salt particles to form a porous layer around the fiber template (Figure 1d). The final bilayer tubular scaffold retaining fibrous morphology at the surface of the innermost layer was obtained after removing the core template by dissolving in distilled water.

**FIGURE 2:**
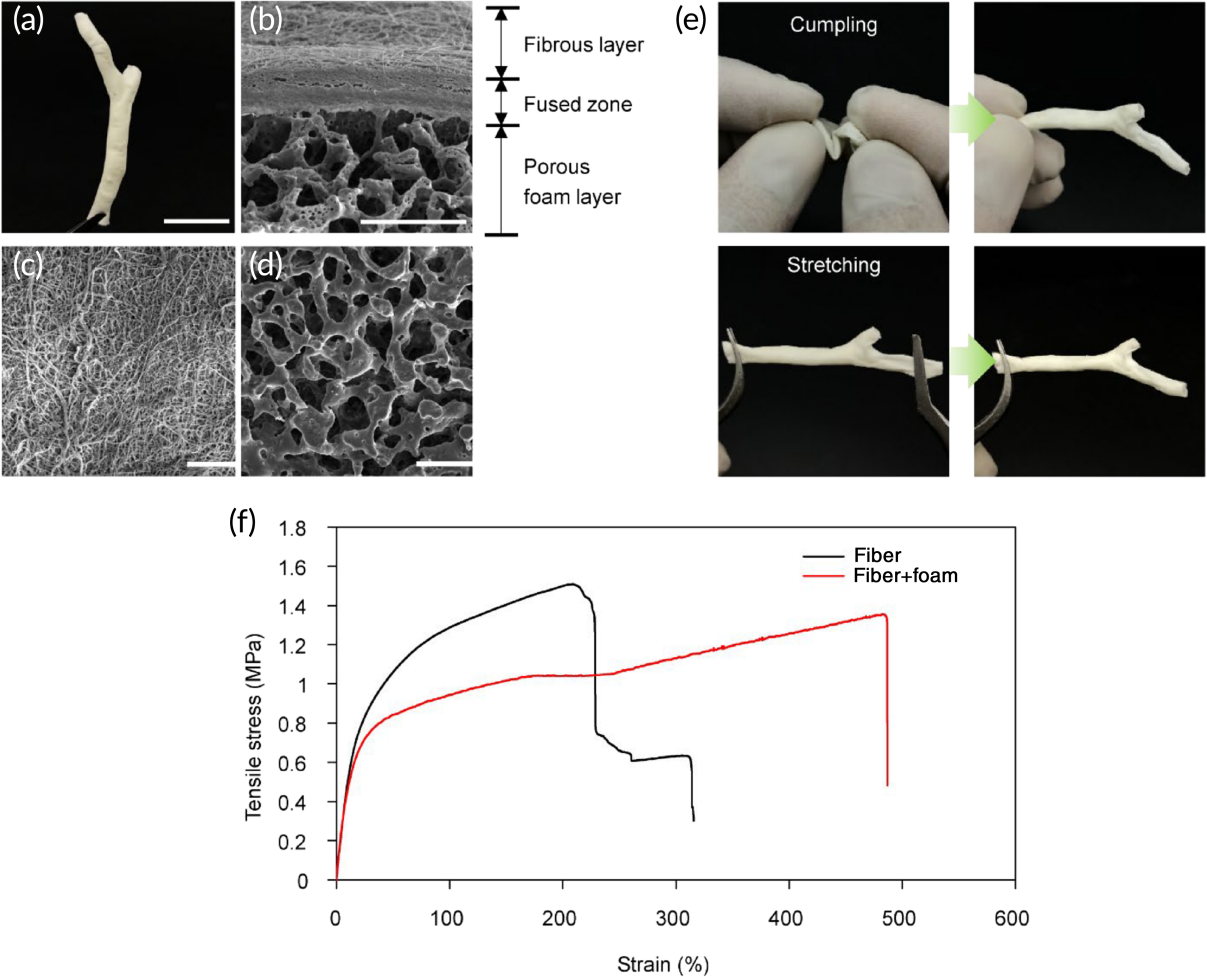
Characterization of 3D customized dual‐layer tubular scaffold. (a) Overall shape of the final scaffold. (b) Representative scanning electron microscopy (SEM) image of cross section through dual layer scaffold. (c) SEM image of inner surface of fiber layer. (d) SEM image of outer surface of porous foam layer. (e) Manual resilience tests by harsh crumpling and stretching. (f) Tensil testing of 3D scaffolds engineered as a dual layer or fiber‐only layer. Scale bars: 20 mm (a); 50 μm (b), 100 μm (c, d)

The correct shape of the extracellular biliary tree was obtained by processing 3D CAD data from medical imaging of rabbit body. The final 3D bilayer tubular scaffold contained a whole anatomical structure composed of CBD and two bifurcated intrahepatic ducts (Figure 2a). Scanning electron microscopy (SEM) was employed to visualize the bilayer organization of the tubular scaffold as illustrated in Figure 2. At the step of dip‐coating on the fiber‐deposited template, the solvent was slightly fusing with the superficial part of the fiber‐coating template thereby forming a fused zone binding the fibrous and porous foam layers (Figure 2b). Due to this rigid fusion, there was no delamination in the bilayer structure which ensured an adequate mechanical strength. The innermost surface of the tubular scaffold was composed of the electrospun fibers (Figures 1c and 2c) while the outer layer of the scaffold retained the salt‐leached porous morphology (Figure 2d). According to the size of used salt particles, the pore sizes were about 50 μm which allowed to mechanically support the fragile and easily collapsing fibrous layer, while maintaining structural flexibility. The fabricated bilayer tubular scaffold could maintain lumen and endure harsh manual manipulations taking place during surgery (Figure 2e). To demonstrate mechanical stability, the scaffolds were loaded on a tensile tester. The results confirmed that the bilayer specimens composed of fiber and foam layers exhibited greater flexibility with a slightly lower elastic modulus and a much longer elongation at break than single‐layer samples (Figure 2f; Figure S1a,b). The bilayer organization could be easily seen at the cross section of tube wall as illustrated in Figure 2b by SEM.

### 2.2 Generation of hepatic progenitors and differentiation toward cholangiocytes

To meet high demand of functional biliary epithelial cells for populating ABD constructs for medical applications, we utilized our recently developed methodology designed to obtain a sizable population of hepatic progenitor cells (hCdHs) with high efficiency, purity, and experimental ease‐of‐use.^14^ The hCdHs have documented potential for self‐renewal and multi‐lineage differentiation and can be rapidly established from the patient‐specific human liver biopsies providing an efficient cell‐based tool for bioengineered practices.

**FIGURE 3:**
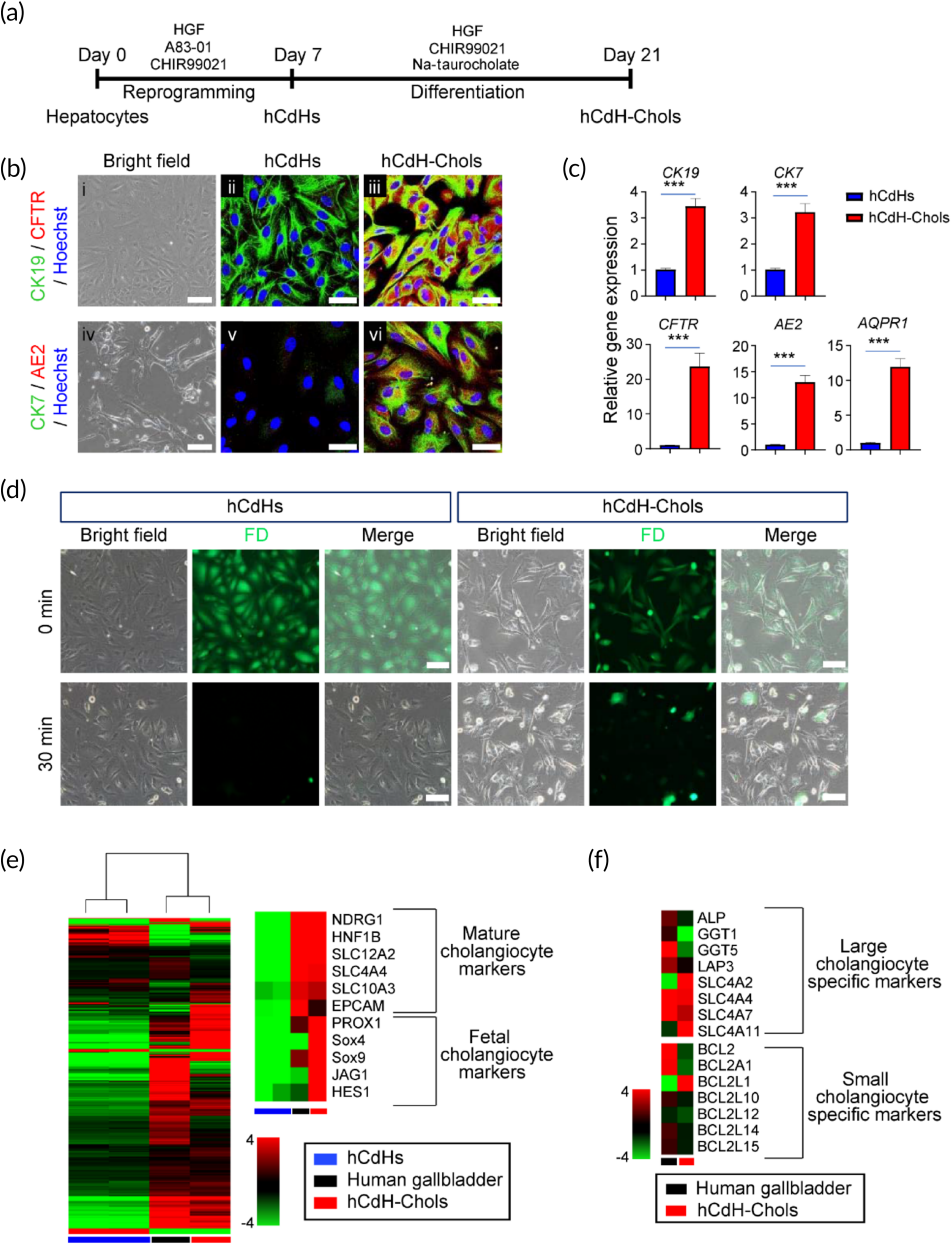
Differentiation of human chemically derived hepatic progenitor cells (hCdHs) toward biliary epithelial lineage in 2D culture. (a) Experimental design. (b) Representative phase contrast (i and iv) and double immunofluorescence (ii, iii, v, vi) images of staining for biliary markers CK19/CFTR, and CK7/AE2 at 14 days after induction of cholangiocytic differentiation as compared to undifferentiated hCdHs used as negative controls. Nuclei were counterstained with Hoechst 33342. Scale bars: 100 μm (i and iv), 50 μm (ii, iii, v, vi). (c) Reverse transcription quantitative real‐time polymerase chain reaction (RT‐qPCR) analysis of biliary marker genes at 14 days post differentiation. GAPDH was used as internal control. Data were analyzed by two‐tailed *t*‐test and presented as means ± *SEM* of three individual experiments performed in triplicates. ***p < 0.001 (d) Representative fluorescence images demonstrating secretion of fluorescein diacetate (FD) indicative of bile acid transfer. Scale bar: 100 μm. (e) Heat map and unsupervised hierarchal cluster analysis of RNA‐seq data generated for human gallbladder tissue, hCdHs, and hCdH‐Chols. (f) Heat map diagram showing differential expression pattern of known markers for large and small cholangiocytes in human gallbladder and hCdH‐Chols. The color bars in panels (e) and (f) indicate gene expression in log2 scale with red being the highest and green the lowest value

To generate hCdHs, adult human hepatocytes were isolated from healthy donor liver tissue (Table S1) and directly reprogrammed into bipotent progenitor cells by a combined treatment with two small molecules A83‐01 and CHIR99021 in the presence of hepatocyte growth factor (HGF).^14^ When hCdHs reached a passage 2, they were subjected to a cholangiocyte differentiation protocol^7^ as outlined in a schematic overview (Figure 3a). The hCdHs cultured for 2 weeks on gelatin‐coated dishes in cholangiocyte differentiation medium (CDM)^7^ containing Na‐taurocholate and CHIR99021 exhibited a marked induction of key structural (CK7, CK19) and functional (CFTR, AE2, AQPR1) biliary markers (Figure 3b,c). The hCdH‐derived cholangiocytes, thereafter referred to as hCdH‐Chols, were also capable to metabolize fluorescein diacetate consistent with acquisition of functional maturity (Figure 3d). Global gene expression analysis confirmed transcriptional changes toward biliary phenotype. The hCdH‐Chols clustered closer to human gallbladder than to undifferentiated hCdHs and expressed mature (*NDRG1*, *HNF1B*, *SLC* families and *EPCAM*) and fetal biliary markers (*PROX1*, *SOX4*, *SOX9*, *JAG1*, and *HES1*) (Figure 3e). Overall, hCdH‐Chols acquired a greater degree of differentiation toward the large (*ALP*, *GGT1*, *GGT5*, *LAP3*, and *SLC* families) rather than small (*BCL* families) cholangiocytes (Figure 3f) considered to be less mature progenitor‐type cell.^15^

**FIGURE 4:**
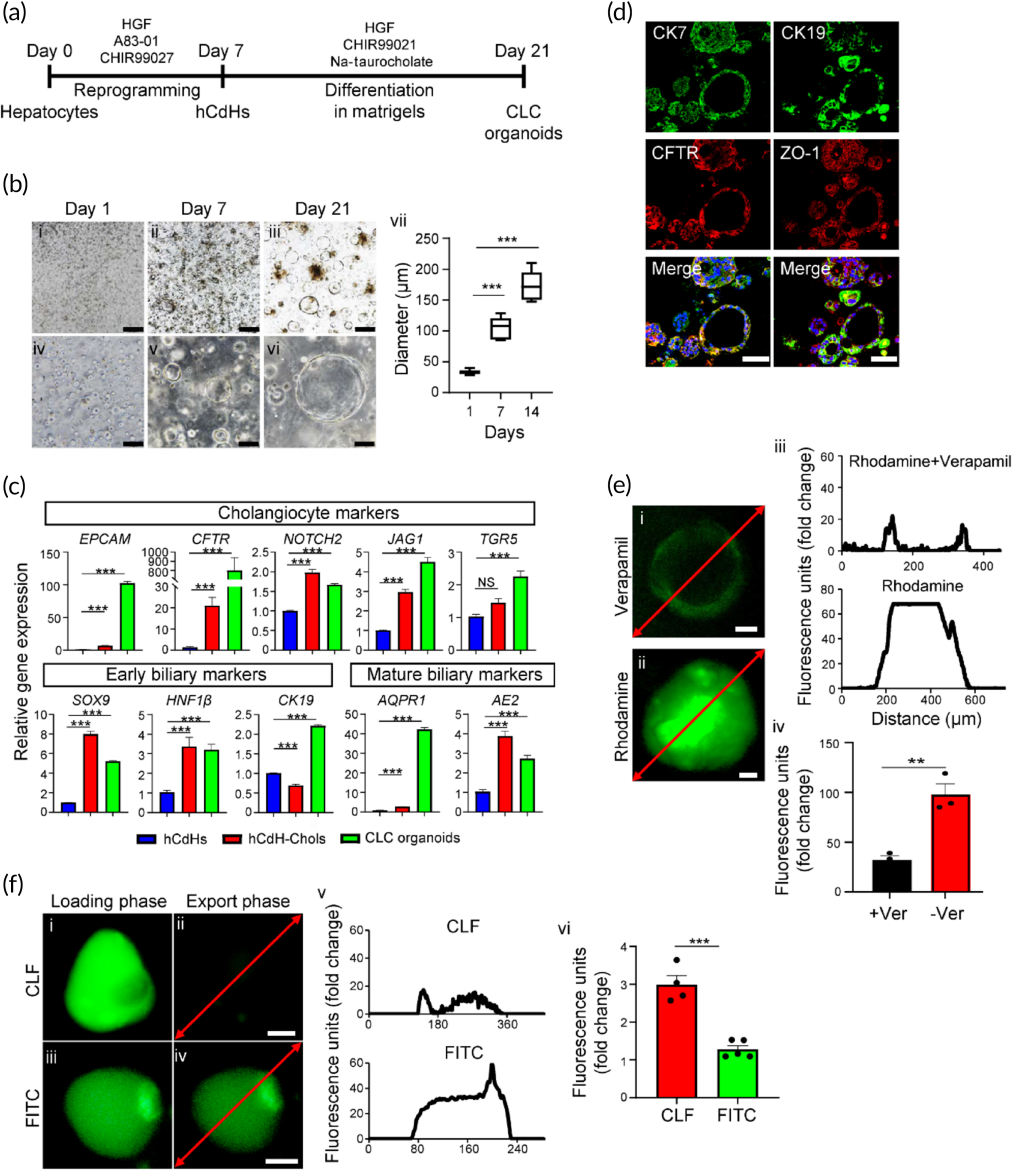
Differentiation of human chemically derived hepatic progenitor cells (hCdHs) toward biliary epithelial lineage in 3D organoid culture. (a) Schematic of the protocol used to generate hCdHs derived cholangiocyte‐like cell (CLC) organoids. (b) Representative phase contrast images of organoids on day 1, 7, and 14. Scale bars: 500 μm (i–iii), 100 μm (iv–vi). Changes in organoid size (vii) are shown as a box plot of means ± *SEM*, ***p < 0.001 (two‐tailed *t*‐test) (*n* = 5). (c) Reverse transcription quantitative real‐time polymerase chain reaction (RT‐qPCR) analysis of the indicated biliary cell marker genes in hCdHs and hCdH‐derived hCdH‐Chols differentiated toward cholangiocytes in 2D and 3D cultures for 14 days. Data are presented as means ± *SEM* of three individual experiments performed in triplicates (*n* = 3). ***p < 0.001 (two‐tailed *t*‐test). NS: not significant. (d) Representative images of double immunofluorescence staining with CK7, CFTR, CK19, and ZO‐1 antibodies. Scale bars: 50 μm. (e) Representative fluorescence confocal microscopy images demonstrating MDR1‐dependent transport of rhodamine 123 into the lumen of hCdH‐Chol organoids and its blockage by the MDR1 inhibitor verapamil (i and ii). Scale bar: 20 μm. Plot profiles of intraluminal fluorescence intensity along the area indicated by red line (iii) and mean intraluminal fluorescence intensity normalized over background (iv). Data are means ± *SEM* (*n* = 3–5). **p < 0.01 by two‐tailed *t*‐test. (f) Representative fluorescence confocal microscopy images demonstrating export of a synthetic bile acid analog cholyl‐lysyl‐fluorescein (CFL) from the lumen of CLC organoids (i–iv). Note that the control organoids loaded with fluorescein isothiocyanate (FITC) retained bright‐green fluorescence (iii and iv). Scale bars: 33 μm (i and ii), 20 μm (iii and iv). Plot profiles of the intraluminal fluorescence intensity (v) in organoids and mean intraluminal fluorescence intensity normalized over background (vi). Data are means ± *SEM* (*n* = 3–5). ***p < 0.001 by two‐tailed *t*‐test

To extend these results to 3D level, we then generated the cholangiocyte‐like cell (CLC) organoids derived from hCdHs as a model system to study functionality of hCdH‐Chols (Figure 4a). The results demonstrated that hCdHs grown three‐dimensionally in Matrigel and CDM rapidly formed typical cyst‐like structures which progressively increased in size within 2 weeks (Figure 4b). The following reverse transcription quantitative real‐time polymerase chain reaction (RT‐qPCR) analysis and immunofluorescence staining confirmed that 2‐weeks‐old organoids expressed marker genes and proteins characteristic for cholangiocyte differentiation (Figure 4c,d). Subsequently, we employed rhodamine 123, a substrate for cholangiocyte surface glycoprotein multidrug resistance protein‐1 (MDR1), to investigate the bile acid transport activity in hCdH‐Chols organoids. The fluorescent rhodamine 123 was efficiently excreted into the lumen of cystic organoids, and its export was blocked by the MDR1 inhibitor verapamil, confirming that the hCdH‐Chols grown three‐dimensionally were functionally proficient in MDR1‐dependent transfer of rhodamine 123 (Figure 4e). Furthermore, when organoids were incubated with a synthetic bile acid analog cholyl‐lysyl‐fluorescein, binding the apical salt and bile transporter, no fluorescence was detected in the lumen of CFL‐loaded organoid after 30 min in contrast to control organoids loaded with the fluorescein isothiocyanate which retained green fluorescence (Figure 4f). These results demonstrate that hCdH‐Chols display biochemical and functional properties analogous to the biliary epithelium in vivo.

### 2.3 Optimization of fibrous scaffold morphology for enhanced cholangiocyte differentiation

In the field of tissue engineering, the electrospun mats typically act as extracellular‐mimicking matrices providing topographic guidance for the seeded cells.^16^, ^17^ Given the central role of integrin‐based adhesion in regulating cellular behavior and nanoscale organization of focal adhesion structures,^18^ many topographical features of the early scaffolds have been designed using nanoscale configuration.^19^, ^20^ However, the slow perfusion mass transport and limited ability to re‐create the complexity and hierarchy of the extracellular matrix structures characteristic of native tissues deter the use of nanoscale‐engineered approaches for clinical applications.^21^, ^22^, ^23^, ^24^, ^25^ To address these issues, we determined the impact of electrospun sheet scaffold on differentiation properties of hCdH‐Chols. For this purpose, hCdHs were resuspended with the same density (10^6^ cells/cm^2^) in reprogramming medium^14^ and plated either onto a standard 2D dish or electrospun fiber scaffold. After 2 days, the medium was replaced to CDM to induce cholangiocyte differentiation for 14 days (Figure S2a). The results of live/dead assay showed that the differences in growth conditions did not affect cell viability. At the end point of experiment, hCdH‐Chols maintained on the electrospun fiber scaffold exhibited a comparable or slightly higher viability than cells cultured on flat dishes (Figure S2b). However, the fiber sheet scaffold provided a more favorable microenvironment for biliary cell differentiation and caused a stronger expression of key marker genes *SSRT2*, *SCR*, *JAG1*, *TGR5*, *CK7*, *AE2*, *CFTR*, *CK19*, and *AQP1* (Figure S2c). Consistent with this, the growth rate and stemness features of hCdH‐Chols cultured on fibrous scaffold were markedly reduced as judged by RT‐qPCR analysis of proliferation (*Ki67*) and progenitor (*CD90*, *EPCAM*) marker gene expression (Figure S2c).

**FIGURE 5:**
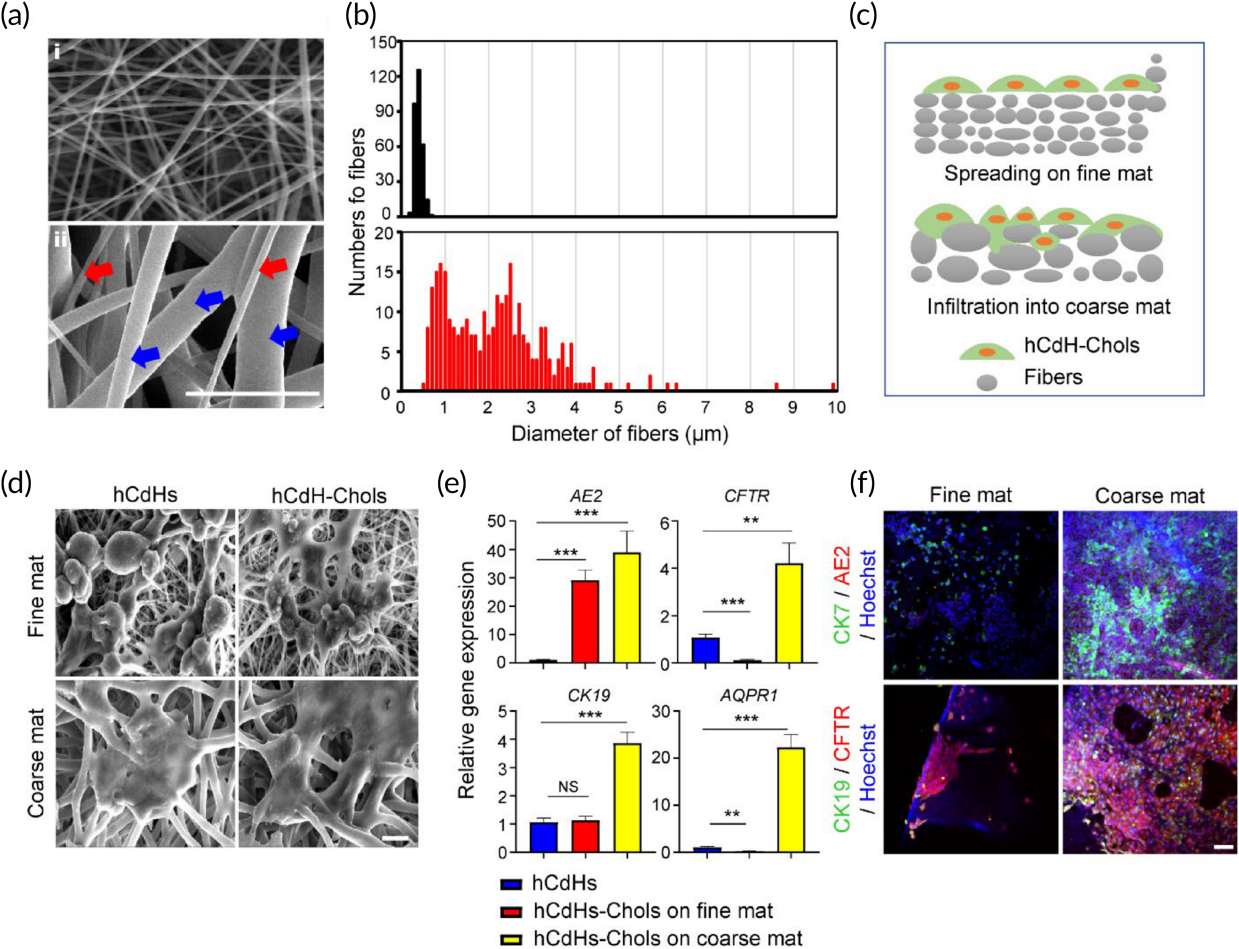
Impact of fiber architecture on functional properties of hCdH‐Chols. (a) Representative scanning electron microscopy (SEM) images of nanofiber only mat (i) and hybrid nano/microfiber mat (ii). Red arrow, nanofibers; blue arrows, microfibers. Scale bar: 10 μm. (b) Distribution of fiber diameters (*n* = 300 fibers for each mat). (c) Schematic diagram illustrating more intimate cell‐to cell and cell‐to matrix interactions within the hybrid nano/microfiber mat. (d) Representative SEM images of human chemically derived hepatic progenitor cells (hCdHs) and hCdH‐Chols seeded on fine and coarse mats and cultured for 14 days in reprogramming and cholangiocyte differentiation media, respectively. Scale bar: 10 μm. (e) Reverse transcription quantitative real‐time polymerase chain reaction (RT‐qPCR) analysis of biliary marker genes *AE2*, *CFTR*, *AQP1*, and *CK19* in hCdH‐Chols cultured on fine and coarse mats. GAPDH was used as internal control. Data were analyzed by two‐tailed *t*‐test and presented as means ± *SEM* of three individual experiments performed in triplicates. **p < 0.01, ***p < 0.001. (f) Representative images of double immunofluorescence staining for biliary marker proteins CK19/CFTR, and CK7/AE2. Scale bar: 50 μm

To extend these data, we then evaluated the effects of fiber sheet architecture on the functional properties of hCdH‐Chols. Given that during typical electrospinning process, the concentration and resulting surface tension of polymer solution define the distribution range of fiber diameters,^16^ we tested the impact of low and high (10% and 18% w/v, respectively) concentrations of PCL solution on fiber dimensions. Under low concentration of PCL, fiber diameters were narrowly distributed within 1 μm (Figure 5a, i). In comparison, a high concentration of polymer solution generated much thicker fibers with diameters ranging from 500 nm to 5 μm or higher, thus including both nanofibers (red arrow) and microfibers (blue arrow) (Figure 5a, ii). SEM of hCdHs and hCdH‐Chols cultured on fine and coarse mats showed that the coarse mat composed of multiscale fibers provided a more intimate cell‐to‐cell and cell‐to‐matrix interactions due to the enlarged microscale pore network, as compared to the sub‐micrometer fine mat (Figure 5b–d). hCdHs grown on a nanofiber‐only fine mat, did not expand into the fiber mesh, and survived on the surface as single cells with minimal cell‐to‐cell interactions. In contrast, when plated on the multiscale coarse mats, hCdHs and in a particular hCdH‐Chols formed considerably larger clusters which were better integrated within the fiber network (Figure 5c,d). Accordingly, RT‐qPCR and double immunofluorescence analyses established a stronger induction of key biliary markers and a more prominent expansion of hCdH‐Chols onto the coarse mat (Figure 5e,f). Thus, fabrication of the coarse fiber structures composed of micro‐ and nanoscale fibers increased the scaffold porosity and thus greatly improved both the spatial organization of hCdHs and efficiency of their differentiation toward biliary phenotype.

### 2.4 Epithelization of ABD with hCdH‐Chols in vitro

**FIGURE 6:**
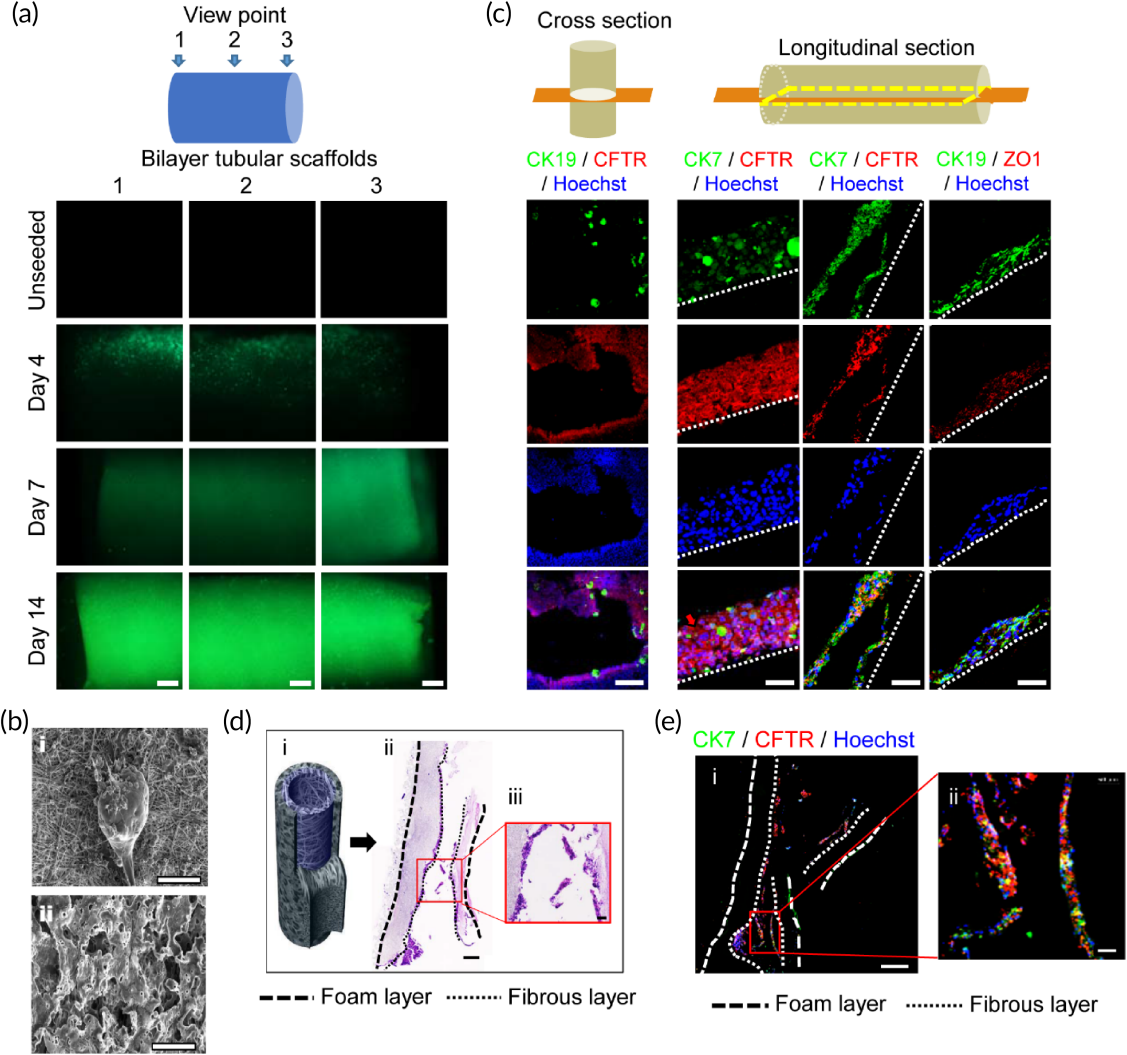
Human chemically derived hepatic progenitor cells (hCdHs) can engraft, survive and differentiate toward biliary epithelial lineage inside artificial bile duct construct. (a, c) Schematic diagrams at the top indicate section orientation. (a) Immunofluorescence images of staining with Calcein‐AM. 3D tubular scaffolds were seeded with hCdHs and cultured in cholangiocyte differentiation medium for 4, 7, and 14 days. Note progressive accumulation of green fluorescence signal through entire inner surface area of 3D scaffold indicative of successful epithelization. Unseeded scaffolds were used as negative control. Scale bar: 500 μm. (b) Scanning electron microscopy (SEM) images show that densely packed cells are lining only inner fiber layer (i) and are absent in outer layer (ii). Scale bars: 200 μm. (c). Double immunofluorescence staining for CK19, CK7, CFTR, and ZO‐1 at 14 days. Scale bars: 250 μm (cross section), 50 μm (longitudinal section). (d) H&E staining of 3D artificial bile duct in vitro. Scale bars: 400 μm (ii), 50 μm (iii). (e) Double immunofluorescence staining with CK7 and CFTR of 3D printed bile duct in vitro using slide scanner. Scale bars: 400 μm (i), 50 μm (ii)

To test whether hCdHs can engraft and survive inside the ABD structures composed of the inner electrospun fibrous layer and surrounded by microporous foam layer for increased stability (Figure 1d), the bilayer tubular scaffolds were loaded with hCdHs and cultured in the medium promoting cholangiocyte differentiation for 14 days. At the selected time points, the scaffolds were stained with calcein‐AM for 30 min and examined under fluorescent microscope (Figure 6a). The results showed a progressive accumulation of green‐calcein fluorescence which was gradually expanding throughout the entire inner surface areas indicative of successful epithelization of tubular scaffolds (Figure 6a). In comparison, the acellular tubular structures used as a negative control did not show any fluorescence signal (Figure 6a).

SEM of each of the two layers of tubular scaffold revealed that hCdHs were attached only to the inner fibrous layer of ABD where they formed a multilayer cell coating encompassing the fibers (Figure 6b, i) and were totally absent in the outer layer (Figure 6b, ii). Morphological and double immunofluorescence analyses of cross and longitudinal sections via inner fibrous layer confirmed that the engrafted cells were organized as densely packed sheets of cells which retained commitment to biliary epithelial differentiation as evidenced by hematoxylin and eosin (H&E) staining and coexpression of biliary (*CK7*, *CK19*, *CFTR*) and tight junction‐associated (ZO‐1) marker proteins (Figure 6c–e). Together, these data demonstrate that the randomly spun mix of nano‐ and microfibers lining the inner surface of the ABD provides a favorable microenvironment for engraftment, proliferation, and differentiation of hCdHs toward biliary lineage cells.

### 2.5 In vivo application

**FIGURE 7:**
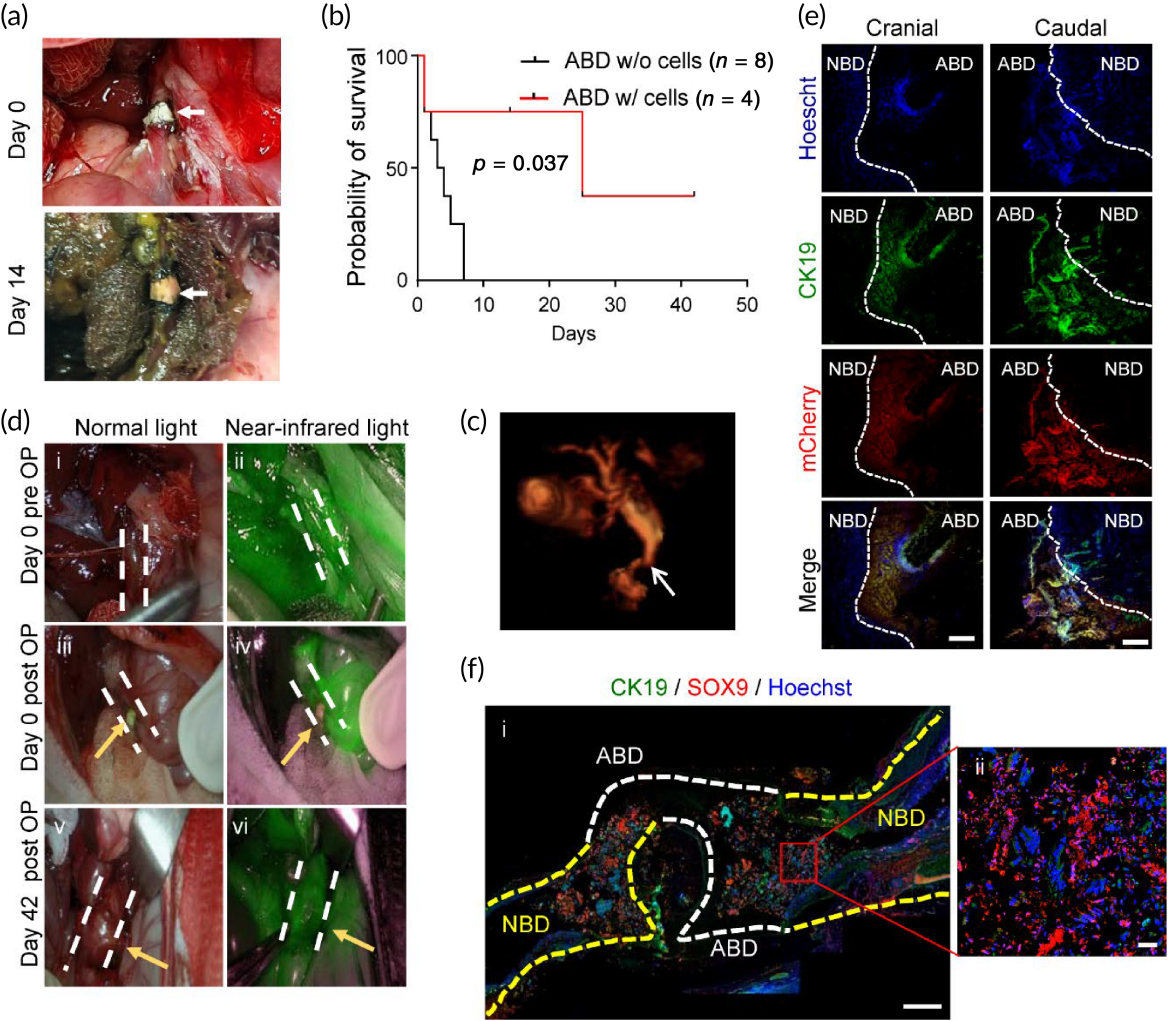
In vivo transplantation of artificial bile duct (ABD) loaded with hCdH‐Chols. (a) Photographs of end‐to‐end anastomosis between artificial bile duct (ABD) graft and native common bile duct (NBD) taken immediately after and 2 weeks after surgery. White arrow, ABD graft. (b) Kaplan–Meier survival analysis. Rabbits receiving cell‐laden ABD implants were monitored for 42 days. (c) Magnetic resonance imaging (MRI) imagining of hepatobiliary tree performed at 14 days postimplantation. White arrow, anastomosis site. (d) Gross observation of abdominal cavity from duodenum site and fluorescent images under near‐infrared light taken after indocyanine green (ICG) injection into auricular vein in normal healthy rabbit (i and ii), immediately after operation (iii and iv) and at 42 days after operation (OP) (v and vi). White dash lines define rabbit bile duct. (e) Double immunofluorescence staining of bile duct anastomosis with CK19 and mCherry antibodies. Cranial means gallbladder‐common bile duct connection and caudal means common bile duct‐duodenum connection. White dash lines show interface between NBD and ABD. Scale bar: 250 μm. White dash lines show interface between NBD and ABD. Scale bar: 250 μm. (f) Double fluorescence staining with CK19 and SOX9 antibodies of the reconstructed bile duct sectioned through duodenum and liver side. Yellow and white dash lines define NBD and ABD, respectively. Scale bars: 1000 μm (i), 50 μm (ii)

Based on the promising results in vitro, we then explored the feasibility of bioengineered bile ducts to restore a CBD defect using rabbit model as a proof‐of principle study. A defect was created by excision of 5‐mm‐long segment from the native healthy CBD and replaced with a 10 mm fragment cut off the full artificial CBD structure populated with hCdH‐Chols which were tagged with mCherry for easy tracing (Figure S3). Despite the small wall thickness (440–470 μm range), the net‐shaped object possessed a sufficient mechanical strength and flexibility to withstand handling and suturing to both anastomosed ends of the original CBD (Figure 7a).

In total, in vivo experiments were carried out on 13 rabbits, including 8 control animals which received acellular ABD grafts and all died within the first 5 days postimplantation (Figure 7b). In comparison, four out of five rabbits implanted with the cell‐containing grafts survived the reconstructive surgery up to the 42nd day, the end point of observation, and only one died the next day due to the postsurgery complications related to the highly complex anatomy and rich blood supply to the biliary tree. Four surviving rabbits appeared clinically well, and from day one moved, ate and behaved normally ([Link], [Link]) demonstrating biological safety and remarkable functional performance of the bioengineered bile duct constructs (Figure 7c). The follow‐up observation period in rabbits with successful ABD transplantation varied and was terminated at 14‐ and 42‐days for implant recovery, and histological and functional assessment. Blood chemistry was performed before and at different time points after transplantation.

During the postimplantation period, most of the serum parameters of liver function remained within the reference range (Table S2 and Figure S4c). A slight increase of biliary enzymes GGT, ALT, and AST observed at 2 weeks post‐ABD implantation returned to the basal levels by the 42nd day. The total bilirubin was similarly reduced to the preimplantation level indicating that there was no obvious damage or toxicity in the native CBD.

During autopsy performed at 2 weeks after surgery, the graft retained its place and appeared as an integral part of the CBD (Figure 7a,b). Despite some yellowing at the lesion site, the bile flow through the anastomosis site was maintained as judged by the postoperative 3D magnetic resonance cholangiography (MRC; Figure 7c; Movie S4). The MRC did not reveal any abnormal bile fluid accumulation besides a slight dilation of the native duct at the anastomosis site (Figure 7c). The free fluid passaging of the indocyanine green compound injected via the auricular vein provided additional evidence that there was no obstacles or blockage of rabbit bile duct at 42 days postimplantation (Figure 7d). The subsequent histological evaluation of the cranial and caudal part of the anastomosis sites confirmed that the transplanted ABD construct was well‐connected to a native rabbit CBD (Figure S4a). Double immunofluorescence staining showed that mCherry‐tagged hCdH‐Chols populating the tubular scaffold expressed lineage‐specific marker proteins CK19 and CK7 (Figure 7f; Figure S4b).

To further illustrate a successful integration of ABD with the native bile duct (NBD), a large segment of liver—duodenum—was isolated at 42 days after implantation and sectioned for histological and immunofluorescent evaluation. The H&E staining confirmed that the anastomosis site was free of biliary stricture thereby allowing an open bile passage into intestine (Figure 7e; Movie S5). We then costained the resected specimen with SOX9 and CK19, two classical markers of biliary differentiation. As expected, only the ABD implant displayed a strong immunoreactivity with a human‐specific SOX9 antibody (Figure S4d) clearly demarcating the end‐to‐end anastomosis from the NBD and revealing the continuity of biliary lining throughout the reconstructed bile duct (Figure 7f). Together, these results establish that our patient‐specific bile duct bioengineered with chemically reprogrammed and microtopographically differentiated cells can successfully restore the large‐scale defect in the rabbit CBD and thus represent an exciting novel candidate for clinical development.

## 3 CONCLUSIONS

The overall objective of this study was to fabricate and evaluate an implantable bioengineered bile duct construct for medical applications. To test this objective, we (i) optimized our published strategy for scaffold fabrication based on 3D design, 3D printing, and postprocessing; (ii) demonstrated the utility of our hCdHs as a compelling source of patient‐specific progenitor cells suitable for epithelization of the ABD structures; (iii) defined the best‐fit scaffold architecture for engraftment, proliferation, and differentiation of hCdHs toward biliary lineage; and (iv) assessed the feasibility of fabricated constructs for replacement therapy in animal studies.

To achieve optimal microtopographic characteristics, the tubular scaffolds were engineered as a synergistic combination of two layers. The inner electrospun fibrous layer was surrounded by outer microporous foam layer that increased the tensile elongation at the break points about 500% reinforcing mechanical durability. Furthermore, we used highly viscous PCL solution to yield fibers with a wide spectrum of diameters ranging from 500 nm to several micrometers. The multiscale architecture of coarse fiber scaffold increased the porosity and permeability thereby improving mass transport. It also offered a more instructive 3D microenvironment that was essential for enhancing cell–cell and cell–matrix interactions acting to promote the physiological cell anchorage and enhance lineage‐specific differentiation of hCdHs toward cholangiocytes as evidenced by SEM, immunofluorescence, and RT‐qPCR analyses. Gene expression profiling, as well as biochemical and functional assessment of bile acid transport activity confirmed that the hCdH‐derived cholangiocytes (hCdH‐Chols) grown in 2D and 3D cultures acquired biliary phenotype. Similarly, the hCdHs plated inside the tubular scaffold survived, expanded, and achieved a high degree of biliary differentiation.

The feasibility of fabricated structures for reconstructive surgery has been demonstrated in a rabbit model of acute large‐sized injury of CBD. For in vivo transplantation studies, the 3D anatomically customized ABD structures were designed from microcomputed tomography images to conform to the precise form and dimensions of the rabbit CBD. The outcomes showed that the bilayer architecture of the ABD promoted the functional maturation of human progenitor cell toward biliary epithelium while providing mechanical stability during surgery. The postoperative 3D MRC confirmed that the bioengineered construct retained a 3D architecture without breakage or deformation and re‐stored the bile flow by the 42nd day, the end point of observation. Animals which received the acellular tubular scaffolds did not survive the replacement surgery. Thus, we believe that our design concept may provide a novel treatment option for the patients with biliary tract disease.

## 4 MATERIALS AND METHODS

### 4.1 Fabrication of 3D customized bilayer tubular scaffold

The overall procedures for scaffold fabrication included the 3D printing of sacrificial template, the fibrous coating of electrospinning, the dip coating of polymer solution, and the removal of template and particles. For the 3D template, commercial water‐soluble filaments of PVA (Filament, ESUN) were printed on a tailor‐made 3D printer driven in the manner of material extrusion. Using the open‐source software, Cura, G‐code for printing path was generated. The other process parameters of nozzle inner diameter, nozzle temperature, and layering thickness were 0.25 mm, 170°C, and 0.15 mm, respectively. To exclude the surface roughness of the as‐printed template, it was immersed and sonicated in warm distilled water at 50°C for 1 min. The detailed information is included in the previously published paper.^13^ For the next step, the electrospinning of polycaprolactone (PCL; MW 80,000; Sigma‐Aldrich) solution was performed while rotating the 3D‐printed PVA template. The PCL granules were dissolved in a solvent mixture of methylene chloride and dimethyl formaldehyde (DMF) at a ratio of 3:1. The solution concentrations were 10% and 18% (w/v) for generating the fine and coarse electrospun mats, respectively. In advance of template coating, the two different mats were compared in terms of functionality to induce the cholangiocytes differentiation. After the comparative experiment, the template coating condition was selected as the concentration of 18% (w/v). For the dip coating step, the fiber‐coated template was dipped into a salt‐dispersed thermoplastic polyurethane (TPU; DAELIM chemical) solution. The TPU granules were dissolved in DMF at a concentration of 15% (w/v). Thereafter, salt particles sieved with a 45‐μm mesh were mixed with the as‐prepared TPU solution at a concentration of 400% (w/w). After drying the solvent on the coated part, it was immersed in sonicated water for 2 min to leach out the salt particles. Finally, the core PVA template was removed by immersing in a sonicated water at 50°C for 30 min, thereby obtaining a 3D bilayer tubular construct. To generate entrances for the water penetration, the small parts were cut at the end of the coated template.

### 4.2 Generation of hCdHs and cholangiocyte differentiation

The study was performed according to protocols approved by the Institutional Review Board of Hanyang University, Seoul, Korea (HYI‐16‐229‐3). Human liver tissues were obtained from three donors operated on in Hanyang University Medical Center (Table S1) with the informed patients' consent. Generation of hCdHs was done following a previously published method.^14^ In brief, the primary human hepatocytes were isolated using a two‐step collagenase perfusion and seeded on a collagen‐coated plate (STEMCELL Technologies, BC, Canada) in William's E media (Gibco, CA, USA). The next day, the medium was changed to the DMEM/F12 (Gibco) reprogramming medium supplemented with 20 ng/mL of HGF (Peprotech), 3 μM CHIR99021 (STEMCELL Technologies), and 4 μM A83‐01 (Gibco), thereafter referred to as HAC. The hCdHs were generated within 1 week and considered as passage one. To induce differentiation, we used our recently published protocol.^7^ In brief, 5 × 10^5^ hCdHs at passage 2 were seeded on a gelatin (Sigma) coated dishes in CDM containing DMEM/F12 supplemented for 2 weeks with 1% nicotinamide, 10% FBS, 0.1 μM Dexamethasone, 1% Insulin transferrin selenium (ITS), 75 ng/ml epithelial growth factor, 7.5 μM CHIR and 75 ng/ml HGF and for the last 2 days with 10 μM sodium taurocholate hydrate (Sigma). The medium was changed every 2 days. CLC organoids were generated as described by Shampaziotis et al.^26^ with modifications. hCdHs were suspended in 70 μl of Matrigel (Corning) at a density of 1 × 10^5^. A 50 μl droplet of cell suspension was placed in the in the center of 24‐well plate and overlaid with 500 μl CDM medium after gel was solidified for 10 min at 37°C, and then the medium was changed every 2 days for 14 days. The ImageJ software was used to measure organoid size at 1, 7, and 14 days.

### 4.3 Cholangiocyte differentiation on fiber scaffolds and ABDs

Before cell seeding, scaffolds and bile ducts were cut, washed with ethanol, and sterilized under UV light. After 30 min, the scaffolds and bile duct fragments were coated with 0.1% gelatin (Sigma) and incubated for 30 min at 37°C and 5% CO_2_. Then, excess gelatin was removed and samples were dried out at room temperature for 2 h. 10^6^ cells were resuspended in 10 μl of HAC media and seeded inside the ABD constructs. They were then incubated for an hour at 37°C and 5% CO_2_. Half milliliter of reprogramming media was used per well in a four‐well plate and replaced with CDM media next day. CDM medium was replaced every 2 days for 14 days.

### 4.4 In vivo transplantation

The animal experiments were performed with the permission of the IACUC (Institutional Animal Care and Use Committee number 2018‐0097A) of Hanyang University, and in compliance with the NIH guidelines and the Hanyang University animal research protocol. Semi‐specific‐pathogen‐free female rabbits at 21 weeks of age weighing 3–4 kg were obtained from the DooYeol Biotech (Seoul, Republic of Korea). All animals were housed under standard conditions with a 12‐h light/dark cycle and fed a standard diet during a 4‐week adaptation period. The rabbits were fasted for 12 h before surgery. Under general anesthesia administered by an anesthesiologist, the animals were immobilized in the supine position and laparotomized via a midline incision in the upper abdomen to expose CBD. The lower CBD was transected, and the ABD was anastomosed end‐to‐end to the proximal and distal ends of the CBD with interrupted 10‐0 Ethilon® sutures under light microscopy. All procedures were performed by a microsurgeon. The adequate bile drainage through the anastomosed ABD was confirmed during surgery. The abdominal cavity was irrigated with warm saline before closing. After surgery, immediately, the rabbits were injected 25 mg/kg pain medicine (PACETA, SHIM POOMG), 40 mg/kg antibiotics (ceftezole sodium, SHIM POOMG), and 0.05 mg/kg immunosuppressant (Prograf, ASTELLAS). Then, the rabbits were administered by these medicines twice a day for 7 days—morning: pain medicine, antibiotics, and immunosuppressant; night: pain medicine and antibiotics. Follow the 1 week, the rabbits only were inoculated the antibiotics and immunosuppressant twice a day— morning: antibiotics and immunosuppressant; night: antibiotics. Note that the immunosuppressant was injected to rabbit only at the morning and all these three medicines were administered via auricular vein. For routine histology, the samples from rabbit liver and reconstructed bile duct were immediately placed in 10% neutral buffered formalin and fixed for 24 h at 4°C. The tissues were processed, embedded in paraffin, sectioned at 4 μm, and stained with H&E. Histopathologic examination was performed by trained pathologists.

## AUTHOR CONTRIBUTIONS

**Jaemin Jeong:** Conceptualization (lead); data curation (lead); supervision (equal); writing – review and editing (equal). **Elina Maria Buisson:** Methodology (equal); writing – original draft (equal). **Suk‐Hee Park:** Conceptualization (lead); data curation (lead); validation (equal); visualization (equal); writing – original draft (equal). **Myounghoi Kim:** Data curation (equal); writing – original draft (equal). **Kyojin Kang:** Investigation (equal). **Sangtae Yoon:** Data curation (equal). **Ji Eun Lee:** Methodology (equal). **Young Won Kim:** Methodology (equal). **Nak Kyu Lee:** Conceptualization (equal); data curation (equal). **Mi Ae Jeong:** Investigation (equal). **Bo‐Kyeong Kang:** Validation (equal); visualization (equal). **Seung Bum Lee:** Resources (equal). **Valentina Factor:** Writing – review and editing (equal). **Daekwan Seo:** Data curation (equal); visualization (equal). **Dongho Choi:** Funding acquisition (lead); writing – review and editing (lead). **Hyunsung Kim:** Formal analysis (supporting); visualization (supporting).

## CONFLICT OF INTERESTS

The authors declare no conflicts of interest.

### PEER REVIEW

The peer review history for this article is available at https://publons.com/publon/10.1002/btm2.10252.

## Supporting information


**Movie S1** Rabbit at 1 day after ABD implantation.

**Movie S2** Rabbit at 14 days after ABD implantation.

**Movie S3** Rabbit at 38 days after ABD implantation.

**Movie S4** MRI imaging of extrahepatic bile duct tree after reconstructive surgery of rabbit common bile duct.

**Movie S5** Fluorescence cholangiography of rabbit common bile duct (CBD) before operation.

**Movie S6** Fluorescence cholangiography of rabbit common bile duct (CBD) at day 0 post‐OP.

**Movie S7** Fluorescence cholangiography of rabbit common bile duct (CBD) at day 42 post‐OP.

**Appendix** S1: Supporting information

## Data Availability

The main data supporting the findings of this study are available within the paper and its Supplementary information. The associated raw data are available from the corresponding author on reasonable request.
