# Supplementary material for: Transplantation of patient‐specific bile duct bioengineered with chemically reprogrammed and microtopographically differentiated cells
Source: Bioeng Transl Med. 2021 Sep 3;7(1):e10252. doi: 10.1002/btm2.10252 (PMC8780056; doi:10.1002/btm2.10252)
Supplement: Supplementary file 8 — Appendix S1: Supporting information [file BTM2-7-e10252-s005.docx]

Supporting Information

**Transplantation of patient-specific bile duct bioengineered with chemically reprogrammed and microtopographically differentiated cells**

Elina Maria Buisson^†^, Suk-Hee Park^†^, Myounghoi Kim^†^, Kyojin Kang^†^, Sangtae Yoon, Ji Eun Lee, Young Won Kim, Nak Kyu Lee, Mi Ae Jeong, Bo-Kyeong Kang, Valentina M. Factor, Daekwan Seo, Hyunsung Kim, Jaemin Jeong^*^, Han Joon Kim^*^, Dongho Choi^*^

^†^These authors contributed equally to this work.

^*^Correspondence should be sent to: [jmj1103@gmail.com](mailto:jmj1103@gmail.com) (J.J.); [thicknyh@hanyang.ac.kr](mailto:thicknyh@hanyang.ac.kr) (H.J.K); [crane87@hanyang.ac.kr](mailto:crane87@hanyang.ac.kr) (D.C.)

**Table of contents:**

Materials and methods ------------------- Pages S3-S6

Figures S1-S4 ------------------- Pages S7-S10

Table S1-S4 ------------------ Pages S11-S12

Movie S1-S7 ------------------ Pages S12

References ------------------ Pages S13

**MATERIAL AND METHODS**

**Immunofluorescence staining.** All samples were fixed in 4% paraformaldehyde. 2D and 3D Matrigel cultures were fixed for 20 min at room temperature; scaffolds and ABD were fixed overnight. Staining was done according to the manufacturer’s recommendation. Nuclei were co-stained with Hoechst 33342 (1:10000, Molecular Probes, USA). Cells were imaged using a TCS SP5 confocal microscope (Leica, Germany).

**Hematoxylin and Eosin (H&E) staining**. Histological evaluation was performed on frozen and paraffin-embedded sections. Samples were either frozen in OCT at -20 °C and fixed in 95% alcohol for 5 minutes or fixed in 4% and paraformaldehyde overnight; and then cryo-sectioned or sectioned into 4-5 µm-thick slides.

**Fluorescein Diacetate (FD) assay.** CLC organoids were washed with PBS and incubated with fluorescein diacetate (Sigma) diluted in CDM media at a concentration of 2.5 µg/ml for 15 min at 37℃. After removing FD-containing medium, cells were incubated in fresh medium for additional 30 min. The fluorescence images were taken immediately after incubation in FD medium and after replacing the fresh medium with Hanks Buffer (HBSS).

**Live/dead staining.** Medium was removed and cells were washed with PBS. To tag cells with Calcein (live staining) or Propidium Iodide (dead staining) (ThermoFisher), 1 µl of calcein and 1 µl of proprium iodide were added to 1 ml of media, and cells, scaffolds and artificial bile duct fragments were incubated for 30 minutes at 37°C and 5% CO_2_. The media was then removed and replaced with fresh media. Pictures were taken using a fluorescent microscope.

**SEM imaging.** The ultrastructure of electrospun fibers and cultured cells was visualized using scanning electron microscopy.^1^ The cell-laden scaffolds were washed 3 times with PBS and fixed for 30 min in 5% glutaraldehyde solution containing 0.1 M sodium cacodylate and 0.1 M sucrose. After washing with distilled water for 5 min, samples were slowly dehydrated by serial incubations in 50%, 60%, 70%, 80%, 90%, and 100% ethanol solutions for 5 min each, and then treated with hexamethyldisilazane (HMDS; J. T. Baker, Phillipsburg, NJ, USA) for 15 min. When completely dried, samples were sputtered with Pt until a 20 nm-thick coating was achieved. The samples were imaged via SEM (S-4800; Hitachi, Japan).

**Cholyl-Lysyl-Fluorescein (CLF) assay.** hCdH-derived organoids were incubated with 5 μM cholyl-lysyl-fluorescein (CLF, Corning) for 30 min at 37 °C and then washed with CDM medium 3 times. Fluorescence images were taken 10 min after the 3d wash. Control organoids were incubated with 5 μM fluorescein isothiocyanate (FITC). Fluorescence images were made using a fluorescence microscope after 10 min. Fluorescence in the organoid lumen was normalized over background measured in the surrounding areas and presented as means ± SEM. The experiments were done in triplicate and analyzed by a 2-tailed t-test.

**Rhodamine 123 assay.** CLC organoids were loaded with 100 μM Rhodamine 123 (Sigma) for 5 min at 37 °C and washed with CDM three times. After the 3d wash, the organoids were incubated in CDM medium for additional 30 min at 37 °C. To demonstrate the MDR1 (Multi Drug Resistance Protein 1)-dependent transport of Rhodamine 123, organoids were incubated with 10 μM Verapamil (Sigma) as a control for 30 min at 37 °C and then the experiments with Rhodamin 123 were repeated. Rhodamine 123 fluorescence in the organoid lumen was normalized over the external background. The measurements were done using ImageJ software. Each experiment was done in triplicate. Error bars are means ±SEM, statistical analysis by a 2-tailed t-test.

**Isolation of mRNA and RT-qPCR analysis.** TRIzol reagent (Ambion, Foster City, CA, USA) and chloroform were used to isolate total RNAs. A Transcriptor First Strand cDNA Synthesis Kit (Roche, Mannheim, Germany) was used for reverse transcription of 1 µg of RNA. Oligonucleotide primers with 1 µg of cDNA and 10 µg of quantitative PCR PreMix (Dyne bio, Seongnam, Korea) were used to conduct real-time polymerase chain reaction (RT-qPCR). A total of 40 cycles broken down into 95℃ for 20 seconds and 60℃ for 40 seconds were used for PCR cycles (Table S1).

**Library preparation and transcriptome sequencing.** Total RNA concentrations were calculated by Quant-IT RiboGreen (Invitrogen, #R11490), and integrity values were accessed by TapeStation RNA screentape (Agilent, #5067-5576). Only high-quality RNA with RNA Integrity Number (RIN) greater than 7.0, was used for RNA library construction.

A library was independently prepared with 1ug of total RNA for each sample by Illumina TruSeq Stranded mRNA Sample Prep Kit (Illumina, Inc., San Diego, CA, USA, #RS-122-2101). The initial step of library preparation involved purifying poly‐A containing mRNA molecules by using poly‐T attached magnetic beads. After this step, the purified mRNA was fragmented into small pieces using divalent cations under elevated temperature. The cleaved mRNA fragments were copied to the top strand of cDNA using SuperScript II reverse transcriptase (Invitrogen, #18064014) and random primers, and reverse complemented strand of cDNA was synthesized by using DNA Polymerase I, RNase H, and dUTP. These cDNA fragments underwent the ends repair process by adding a single ‘A’ base and adapter ligation. After these steps, the final cDNA libraries were created and enriched with PCR. The created libraries were quantified using KAPA Library Quantification kits for Illumina Sequencing platforms according to qPCR Quantification Protocol Guide (KAPA Biosystems, #KK4854) and qualified by TapeStation D1000 ScreenTape (Agilent Technologies, # 5067-5582). Indexed libraries were then paired end sequenced by Illumina Hiseq2500 (Illumina, Inc., San Diego, CA, USA) at Macrogen Inc.

**Bioinformatics analysis.** Illumina standard pipeline and real-time analysis (RTA) tools were employed for processing of raw images, base calling and generating FASTQ data by using BCL2Fastq (v2.20, Illumina Inc., San Diego, CA, USA) from paired-end RNA-sequencing data. The 101bp×2 read sequences were preprocessed by using Sickle (v1.33) ^2^ or trimming bad quality sub sequences and CutAdapt (v1.5) ^3^ for removing adapter sequences. After preprocessing, the sequencing reads aligned to hg19 human reference genome (University of California, Santa Cruz provided from Illumina iGenomes, <https://support.illumina.com/sequencing/sequencing_software/igenome.html>) by using RSEM (v1.2.31) with STAR (v2.5.2b) alignment software ^4, 5^*.* The abundance of the expression level of a transcript was measured as the score of TPM (Transcripts Per Kilobase Million) for easier comparison of proportion of reads that mapped to a gene in each sample. The raw sequencing data are available at GEO (https://www.ncbi.nlm.nih.gov/geo) with accession number of GSE152809. Clustering analysis was performed using Cluster3.0 (Michael Eisen lab, http://eisenlab.org/), and visualization was performed by R software (v3.3.2, https:www.r-project.org).

**MRI imaging.** MRI was performed 1 day before and 14 days after surgery using 3T MRI scanner (Ingenia; Philips Healthcare, Best, The Netherlands) with a small extremity coil. The rabbit was anesthetized with intramuscular injection of ketamine (40−100 mg/kg) and xylazine (5−13 mg/kg) during the MRI scan using previously described and validated protocol^6^. Briefly, a three-plane localization imaging gradient echo sequence was obtained first, followed by respiratory-triggered 3D magnetic resonance cholangiopancreatography (MRCP) images and thick-slab single-shot 2D MRCP images were obtained. The acquired 3D MRCP images were saved in digital imaging and communication medicine (DICOM) format and processed into a 3D object in a 3D-printable file format (STL, surface tessellation language) using commercial software (Mimics; Materialise, Louvain, Belgium).

**Blood collection.** Five ml of blood were collected through the marginal ear artery of the rabbit. Three ml of blood were placed into the EDTA- and 2 ml into the heparin-containing test tubes which were kept at 4^0^C for 24 hours. The analyses were performed in animal hospital.

**Indocyanine green (ICG) fluorescence cholangiography**. ICG cholangiography was performed in healthy rabbit, immediately after (0 day) and at 42 days post-operation (one rabbit per each time point). ICG (DONGINDANG, DID indocyanine Green inj, 07280, 25mg) was diluted to 1 mg/ml in sterile normal saline, and two ml were injected to each rabbit via auricular vein. The images were captured by near-infrared light laparoscope (Visera Elite2 ICG system, Olympus) within 5 min.

**Statistical Analysis.** All data are expressed as means ± SD or means ± SE. Each in vitro experiment was repeated at least two to three times as specified in the figure legend. Three technical replicates were used for all measurements. Statistical analyses were performed using paired and unpaired Student’s t-test by Excel 2016. *P < 0.05; **P < 0.01; ***P < 0.001; ****P < 0.0001.

**Supplementary Figures**

**
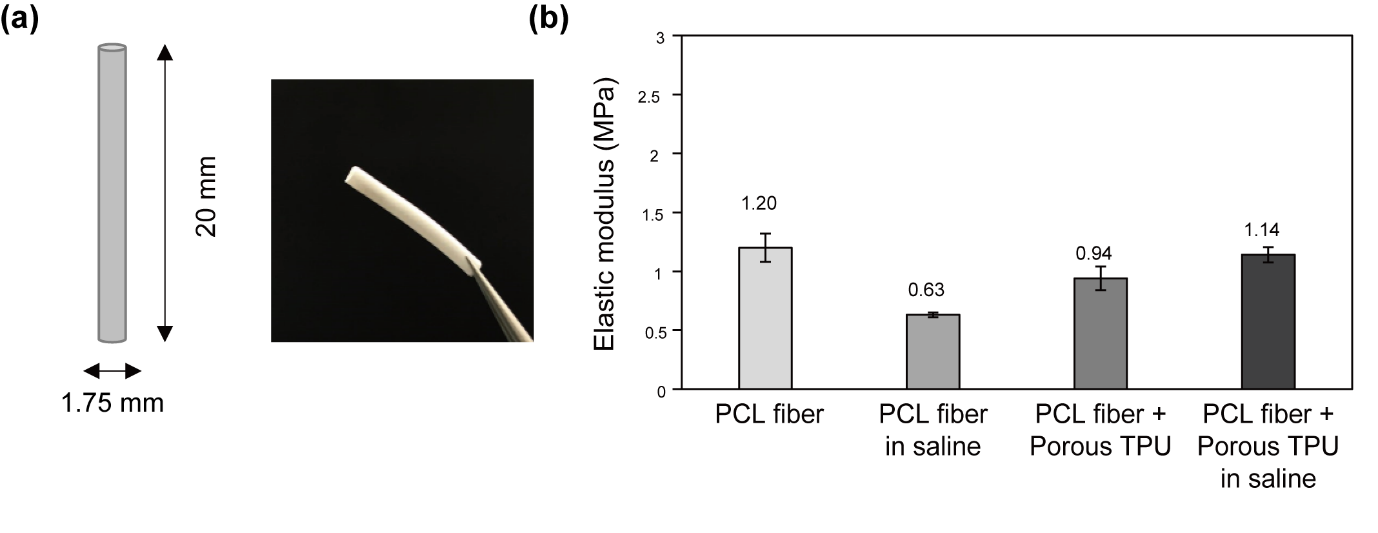
**

**Figure S1.** Comparison of the elastic moduli of single- and dual-layer tubular samples in dry and wet condition (dipping in saline solution). (a) Sample preparation for tensile test of tubular scaffolds. (b) Histogram of elastic moduli of each sample (mean ± standard deviation, n = 3).


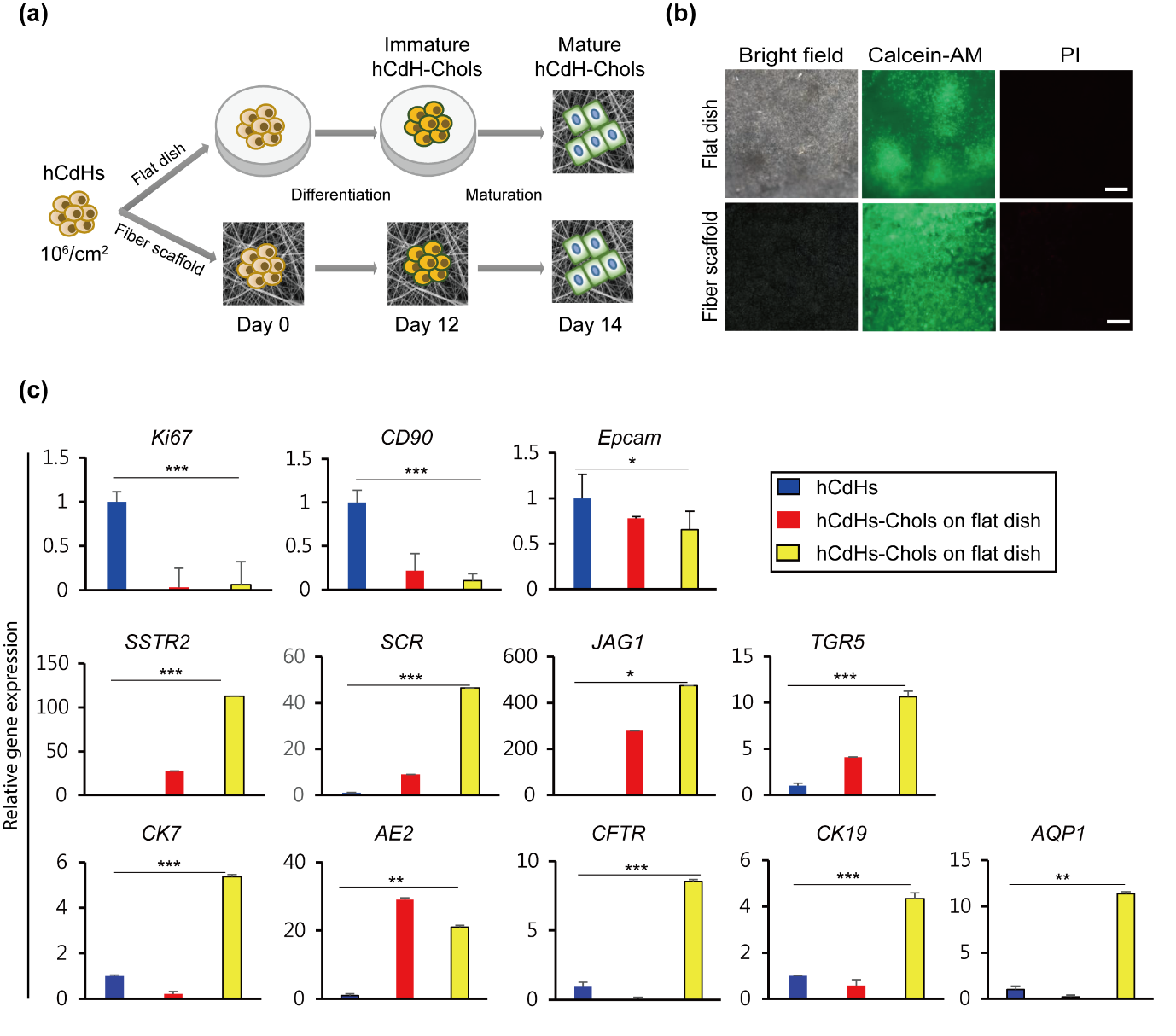


**Figure S2.** Comparison of cholangiocyte differentiation of hCdHs seeded and cultured on flat dishes and fiber scaffolds. (a) Experimental design. (b) Representative phase contrast and Live/Dead staining images using Calcein-AM (Live, green) and Propidium iodine (PI) (Dead, red). hCdHs were plated on flat dishes and fiber scaffold and cultured in cholangiocyte differentiation medium for 14 days. Scale bars: 500 µm. (c) RT-qPCR analysis of proliferative, progenitor and cholangiocyte markers. Non-differentiated hCdHs were used as negative control. GAPDH was used as internal control.


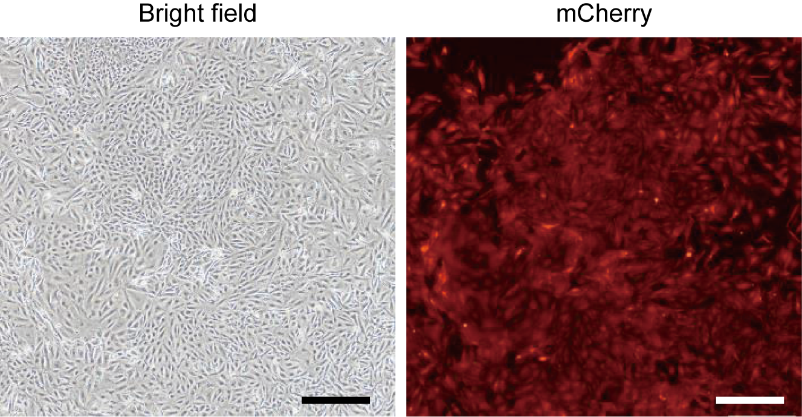


**Figure S3.** Fluorescence staining of mCherry-tagged hCdHs. Bright and mCherry fluorescence images of hCdHs prior to seeding into artificial bile duct. Scale bars: 500µm.


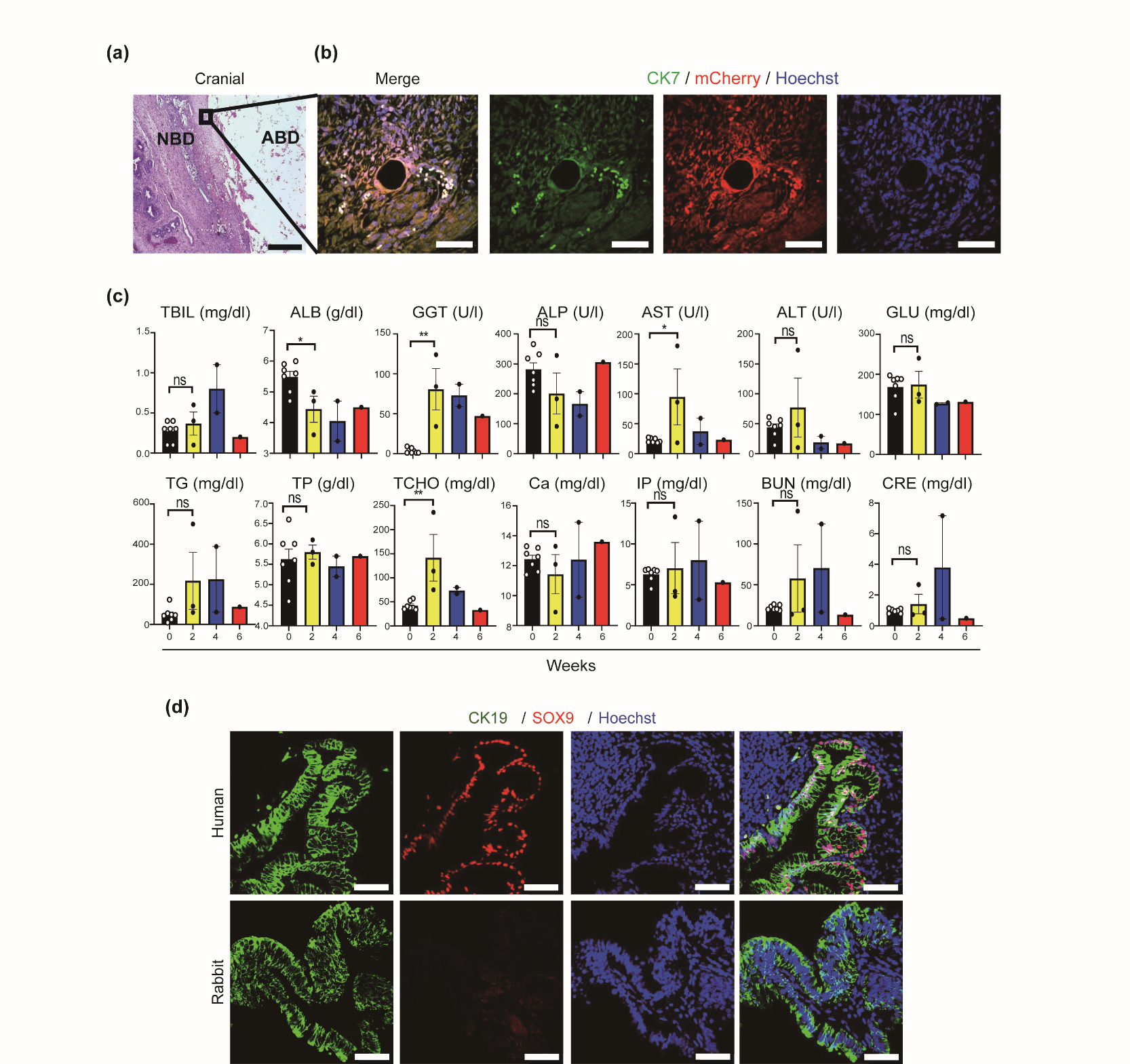


**Figure S4.** hCdH-Chols populating 3D bioprinted artificial bile ducts (ABD) express cholangiocyte-specific markers. (a) H&E staining of anastomosis sites of cranial part. Scale bars: 500 µm. (b) Immunofluorescence staining with CK7 of the boxed area (shown in a) at the interface between the native bile duct (NBD) and ABD graft. Single channel and merged images show that mCherry-tagged hCdH-Chols express a key marker of cholangiocyte differentiation, CK7. Nuclei counterstained with Hoechst. Scale bars: 50 µm. (c) Dynamic changes in blood biochemistry in the rabbit at different time points after ABD implantation. Data are presented as means ± SEM, n=7 (0W) and n=3 (2W). P < 0.01 (**), 2-tailed t-test. (d) Double immunofluorescence staining of rabbit and human gallbladder with CK19 and SOX9. Note that human-specific SOX9 antibody recognized only human whereas anti-CK19 with a broad species specificity was reactive with both rabbit and human tissues.

**Table S1.** Clinical characteristics of liver donors.

| Donor | 1 | 2 | 3 |
| --- | --- | --- | --- |
| Ethnicity | Asian | Asian | Asian |
| Sex | Female | Male | Female |
| Age (years) | 54 | 33 | 35 |
| Body mass index | 24.9 | 23.5 | 19.5 |
| Diagnosis | Intrahepatic Stone | Liver Donor | HCC |
| Operation | Left lateral sectionectomy | Laparo RHH | Wedge resection and cholecystectomy |
| Child-Pugh score | N/A | N/A | 5 (Class A) |
| Albumin (g/dL) | 4 | 4.7 | 3.9 |
| PT(INR) | 1.05 | 0.94 | 1.03 |
| Total bilirubin (mg/dL) | 0.46 | 0.7 | 0.3 |
| ALP (U/L) | 67 | 72 | 100 |
| AST (U/L) | 24 | 16 | 23 |
| ALT (U/L) | 17 | 10 | 16 |
| Hepatitis | None | None | None |
| Alcohol use | N/A | N/A | N/A |
| Smoker | N/A | N/A | N/A |
| Specimen weight (g) | N/A | 0.42 | 1.02 |

PT, prothrombin time; INR, international normalized ratio; ALP, alkaline phosphatase; AST, aspartate aminotransferase; ALT, alanine aminotransferase

Table S2. Changes in hematological parameters after implantation of ABD.

| **Blood Test** | **Reference** | **Units** | **Day 0** | **Day 14** | **Day 28** | **Day 42** |
| --- | --- | --- | --- | --- | --- | --- |
| Total Bilirubin (TBIL) | 0.1 – 0.4 | mg/dl | 0.3 | 0.1 | 0.5 | 0.2 |
| Total Cholesterol (TCHO) | 11 – 74 | mg/dl | 38 | 236 | 67 | 33 |
| Creatinine (CRE) | 0.6 – 1.4 | mg/dl | 1.11 | 0.73 | 0.44 | 0.49 |
| Blood Urea Nitrogen (BUN) | 10.9 – 28.0 | mg/dl | 23.4 | 19.2 | 16.5 | 13.4 |
| Glucose (GLU) | 115 – 214 | mg/dl | 5.6 | 132 | 125 | 131 |
| Triglyceride (TG) | 48 – 205 | mg/dl | 6.0 | 91 | 61 | 87 |
| Inorganic Phosphate (IP) | 1.6 – 4.1 | mg/dl | 49 | 3.6 | 3.2 | 5.3 |
| Calcium (Ca) | 12.5 – 14.5 | mg/dl | 6.8 | 13.3 | 14.9 | 13.6 |
| Total Protein (TP) | 4.9 – 6.9 | g/dl | 12.6 | 6.1 | 5.7 | 5.7 |
| Albumin (ALB) | 4.6 – 6.3 | g/dl | 188 | 4.7 | 4.7 | 4.5 |
| Gamma GT (GGT) | 5 - 18 | IU/L | 9 | 124 | 87 | 47 |
| Alkaline Phosphatase (ALP) | 89 - 535 | IU/L | 271 | 328 | 207 | 306 |
| Alanine aminotransferase (ALT) | 12 - 72 | IU/L | 45 | 173 | 29 | 17 |
| Aspartate aminotransferase (AST) | 9 - 36 | IU/L | 20 | 180 | 15 | 23 |

Table S3. List of primer used for RT-qPCR.

| **Primer** | **Forward Sequence** | **Reverse Sequence** |
| --- | --- | --- |
| CK7 | GAT AAA AGG CGC GGA GTG TC | GAA ACC GCA CCT TGT CGA TG |
| CK19 | TCC GAA CCA AGT TTG AGA CG | CCC TCA GCG TAC TGA TTT CC |
| AE2 | GGG GAA AGT TGA GTT GGG AGA | CTG GCT CTG GCG TAC AGA AA |
| AQPR1 | GGC CAG CGA GTT CAA GAA GAA | TCA CAC CAT CAG CCA GGT CAT |
| CFTR | AGT TGC AGA TGA GGT TGG GC | AAA GAG CTT CAC CCT GTC GG |
| KI67 | GAG GTG TGC AGA AAA TCC AAA | CTG TCC CTA TGA CTT CTG GTT GT |
| CD90 | GTT AGG CTG GTC ACC TTC TG | AGA CTG TTA GCA GGA GAG CGA TGC |
| EPCAM | GAA CAA TGA TGG GCT TTA TG | TGA GAA TTC AGG TGC TTT TT |
| SSTR2 | GAA AAG CAA AGA TGT CAC ACT GGA | TTG GCA TAG CGG AGG ATG AC |
| SCR | TGC TCA CCA GCA GAA ATG GT | AGG TAG GAG TGC CGC TTC TC |
| JAG1 | GGC CGA GGT CCT ATA CGT TG | GCG TCG TCA GCA ATT TCA CA |
| NOTCH2 | ATG GTG GTC AGT GCA TGG AT | GAA AAT CCC GGG GGA CAA CG |
| SOX9 | GAG GAA GTC GGT GAA GAA CG | ATC GAA GGT CTC GAT GTT GG |
| HNF1β | GGG GCC CGC GTC CCA GCA AA | GGC CGT GGG CTT TGG AGG GGG |
| TGR5 | CTG GCC CTG GCA AGC CTC AT | CTG CCA TGT AGC GCT CCC CGT |
| GAPDH | TGC ACC ACC AAC TGC TTA GC | GGC ATG GAC TGT GGT CAT GAG |

Table S4. List of antibodies used for immunofluorescence staining.

| Antibody | Vendor | Catalog No. |
| --- | --- | --- |
| CK19 | Santa Cruz Biotechnology | sc-376126 |
| CK7 | Abcam | ab9021 |
| AE2 | Abcam | 42687 |
| CFTR | Allomone labs | ACL-006 |
| ZO-1 | Abcam | ab96587 |
| SOX9 | Abcam | ab185966 |
| mCherry | SICGEN | AB0040-200 |
| Hoechst 33342 | LifeTechnologies | H3570 |

Movie S1. Rabbit at 1 day after ABD implantation.

Movie S2. Rabbit at 14 days after ABD implantation.

Movie S3. Rabbit at 38 days after ABD implantation.

Movie S4. MRI imaging of extrahepatic bile duct tree after reconstructive surgery of rabbit common bile duct.

Movie S5. Fluorescence cholangiography of rabbit common bile duct (CBD) before operation.

Movie S6. Fluorescence cholangiography of rabbit common bile duct (CBD) at day 0 post-OP.

Movie S7. Fluorescence cholangiography of rabbit common bile duct (CBD) at day 42 post-OP.

**REFERENCES**

1. Patel S, Jung D, Yin PT, et al. NanoScript: a nanoparticle-based artificial transcription factor for effective gene regulation. *ACS Nano*. Sep 23 2014;8(9):8959-67. doi:10.1021/nn501589f

2. Shor L, Güçeri S, Wen X, Gandhi M, Sun W. Fabrication of three-dimensional polycaprolactone/hydroxyapatite tissue scaffolds and osteoblast-scaffold interactions in vitro. *Biomaterials*. Dec 2007;28(35):5291-7. doi:10.1016/j.biomaterials.2007.08.018

3. Martin M. Cutadapt removes adapter sequences from high-throughput sequencing reads. 2011: 17, 3.

4. Dobin A, Davis CA, Schlesinger F, et al. STAR: ultrafast universal RNA-seq aligner. *Bioinformatics*. Jan 1 2013;29(1):15-21. doi:10.1093/bioinformatics/bts635

5. Li B, Dewey CN. RSEM: accurate transcript quantification from RNA-Seq data with or without a reference genome. *BMC Bioinformatics*. Aug 4 2011;12:323. doi:10.1186/1471-2105-12-323

6. Park SH, Kang BK, Lee JE, et al. Design and Fabrication of a Thin-Walled Free-Form Scaffold on the Basis of Medical Image Data and a 3D Printed Template: Its Potential Use in Bile Duct Regeneration. *Acs Appl Mater Inter*. Apr 12 2017;9(14):12290-12298. doi:10.1021/acsami.7b00849
